# Assessment of Mastery Motivation and Neurodevelopment of Young Children at High Risk for Developmental Delays

**DOI:** 10.3390/jintelligence11060115

**Published:** 2023-06-09

**Authors:** Patricia Blasco, Sage Saxton, Lily Marie Gullion, Tun Zaw Oo, Stephen Amukune, Krisztián Józsa

**Affiliations:** 1 Institute on Development and Disability, Department of Pediatrics, School of Medicine, Oregon Health & Science University, Portland, OR 97239, USA; 2 Division of Occupational Science and Occupational Therapy, University of North Carolina, Chapel Hill, NC 27599, USA; 3 MTA-MATE Early Childhood Research Group, 7400 Kaposvár, Hungary; 4 Institute of Education, Hungarian University of Agriculture and Life Sciences, 7400 Kaposvár, Hungary; 5 School of Education, Pwani University, Kilifi 80108, Kenya; 6 Institute of Education, University of Szeged, 6722 Szeged, Hungary

**Keywords:** infants, developmental assessment, early intervention, eligibility, toddlers, mastery motivation, neurodevelopmental status

## Abstract

Young children’s mastery motivation and neurodevelopmental evaluation can contribute to overall early assessment for early intervention evaluation. At present, children born preterm (<37 weeks gestation) and with a low birth weight (LBW; <2500 g) are at increased risk of experiencing developmental delays and more nuanced cognitive and language challenges. The main objective of this exploratory study was to examine the connection between preterm children’s mastery motivation and their neurodevelopment, as well as to determine whether assessing mastery motivation can enhance assessment practices for early intervention (EI) programs. Parents of children born preterm completed the revised Dimensions of Mastery Motivation Questionnaire (DMQ18). Neurodevelopment was measured on the Bayley Scales of Infant and Toddler Development (BSID-III). Results revealed significant correlations between DMQ18 and BSID-III measures. Multivariate analysis showed that infants and toddlers born with a very low birth weight (VLBW; <1500 g) scored significantly lower on the infant DMQ18 and the BSID-III measures. Regression analyses revealed that birth weight and home environment were significant predictors of the children’s eligibility for EI programs. Infants’ social persistence with other children, gross motor persistence, and mastery pleasure, as well as toddlers’ objective cognitive persistence, social persistence with adults, gross motor persistence, mastery pleasure, and negative reaction to frustration, were important markers for evidenced-based practices in EI programs. This study demonstrates the utility of the DMQ18 as a contributory assessment measure and the importance of birth weight and home environment in predicting EI enrollment.

## 1. Introduction

The survival rate of preterm infants increased worldwide in recent years, and their high rate of neurological problems has become a considerable concern for the general public health and human development professionals (Lugli et al. 2020). Medical experts and educators held the belief for a considerable period that low-birth-weight (LBW) or preterm infants who did not have significant cerebral damage would catch up with their peers in terms of development once they became medically stable (French 2017). However, several studies have demonstrated that these children are at a higher risk of developing conditions related to pervasive developmental disorders, experiencing developmental delays, and facing more complex language and cognitive challenges, including deficits in executive function (EF) (Aarnoudse-Moens et al. 2009; Barre et al. 2011; Blasco et al. 2020; Duvall et al. 2015; Guarini et al. 2009; Hutchinson et al. 2013; Lee et al. 2011). Saigal and Doyle (2008) found that most studies of infants with very low birth weight (VLBW, <1500 g) reported ongoing difficulties related to cognitive impairment and academic underachievement.

To help infants with LBW or VLBW overcome different kinds of developmental challenges, neonatal intensive care unit (NICU) follow-up clinics track early development, often up to 3 years of age. Other research has also suggested that preterm infants are more likely to require special education at school age than their full-term peers (De Jong et al. 2012; Griffiths et al. 2019; McGowan 2022). Furthermore, researchers have found later social–emotional difficulties that impact learning and lead to lifelong risks (Linsell et al. 2015; Scott et al. 2017). Therefore, it is necessary to identify preterm children’s developmental delays early and to connect families with necessary early intervention (EI) services (Vohr et al. 2004).

Educators and researchers (Green and Morgan 2017; Hashemi et al. 2017; Huang et al. 2022; Józsa et al. 2017; MacTurk et al. 1995; Morgan et al. 2017a; Wang et al. 2011) have used the revised Dimensions of Mastery Questionnaire (DMQ18) based on observations, structured challenging tasks, and rating scales to determine whether preterm children qualify for/require EI services. Mastery motivation is a powerful latent factor that encourages individuals to achieve their potential (Wang et al. 2011, 2017). Morgan et al. (1990) similarly defined mastery motivation as a strong driving force for individuals to master challenging tasks. Later, Barrett and Morgan (2018) redefined it as “the urge or psychological push to solve problems, meet challenges, and master ourselves and our world” (p. 4). Therefore, it is crucial to investigate the link between the mastery motivation of children who are at high risk of developmental delays and their neurodevelopmental status. In this study, we explore the correlation between mastery motivation and neurodevelopmental status among children at high risk of developmental delays and examine the potential advantages of assessing mastery motivation for EI programs specially designed for these children.

### 1.1. Role of Mastery Motivation for Preterm High-Risk Children

Mastery motivation has been defined as one of the most important aspects of a child’s development, and it is useful for his/her developmental evaluation process. It can stimulate a child’s attempts to master some kinds of difficulties challenging him/her (Green and Morgan 2017). The developmental construct of mastery motivation refers to a child’s ability to independently engage in goal-directed behavior by attempting to master a task that is at least moderately challenging (Morgan et al. 2017a; Borbélyová et al. 2019). Mastery motivation emerges in late infancy and is a major construct in understanding early cognitive and social attempts at problem solving (Keilty et al. 2015). It can readily be assessed in the developmental period (Kelley et al. 2000). 

Mastery motivation is an individual’s driving force to overcome the problems they experience (Wang et al. 2020), and it is an important developmental concept to use in assessing children’s developmental factors (Green and Morgan 2017; Marcineková et al. 2020). Children with developmental delays have demonstrated lower levels of mastery motivation compared to children born full-term (Young and Hauser-Cram 2006). To help these children, mastery motivation has the predictive power of children’s developmental assessment (Józsa and Molnár 2013) and is supported by Wang et al. (2016) finding that mastery task persistence can predict both cognitive and fine motor skills in preterm high-risk children at 6 months of age. Task persistence and mastery pleasure are key components of mastery motivation. Therefore, we assume that mastery motivation assessment can predict preterm high-risk children’s educational development and eligibility for EI. According to Hashemi et al. (2017), preterm high-risk children’s persistence in facing challenges and compliance with their peers/caregivers shows the requirement for emotional self-regulation. Therefore, the role of mastery motivation, which can assess children’s persistence, is of considerable importance for developing and implementing early childhood developmental programs.

In a recent study conducted in Hungary, researchers examined a measure of mastery motivation using the DMQ18 (Borbélyová 2016; Morgan et al. 2020), a parent report questionnaire, and compared scores to traditional academic measures of math, reading, etc. Results for 327 school-age children (M = 5 years of age) showed that children’s negative reactions to failure/challenge as measured on the DMQ18 predicted multiple measures of school performance, over and above the role of child IQ and socioeconomic background. Mastery pleasure as measured on the scale predicted reading, and persistence in peer interaction predicted social skills in the primary grades. These findings demonstrate the importance of motivation and achievement-related emotions in school readiness and school success. Other researchers have found that the measurement of mastery motivation is a useful tool for children with known disabilities, such as cerebral palsy (CP) (Salavati et al. 2018). The authors concluded that the DMQ18 provided helpful findings with respect to different aspects of motivation in children with CP as defined by the parents’ perspectives; hence, the DMQ18 can be used as an intervention tool.

The studies described above highlight the importance of alternative assessments, including mastery motivation, for all populations of children but particularly for those at risk of developmental delay and those with disabilities. Including parent report measures is recommended for both practice and use of informed clinical opinion in eligibility determination to address additional areas of concern (Lucas and Shaw 2012). As such, assessment of the construct of mastery motivation becomes important and may become a useful part of the “toolbox” for EI referral and practice.

### 1.2. Role of Neurodevelopmental Skills for Preterm High-Risk Children

The first years of life from gestation onward represent a period of rapid brain growth and development with fluctuating growth spurts (Kvestad et al. 2022). Therefore, neurodevelopmental skills are very important and frequently assessed in young children with high-risk developmental delays (Jary et al. 2011). Regarding the neurodevelopmental skills of infants and toddlers, research has been conducted in clinical fields investigating multiple infants behaviors, such as visual processing, preferences, motor skills, affective processes, social/playful behaviors, and verbal or non-verbal cognitive skills (Galsworthy et al. 2000; Klein-Radukic and Zmyj 2023; Krogh and Væver 2019). Researchers who study the early development of the brain utilize advanced techniques such as magnetic resonance imaging (MRI) to identify and evaluate neurological outcomes for this group by detecting changes in the brain (Camerota et al. 2022; Miller et al. 2020). 

Preterm children’s cognitive skills include their sensorimotor development, exploration and manipulation, object relatedness, memory, habituation, visual preference, object permanence, and other related aspects (Klein-Radukic and Zmyj 2023). Their language skills are composed of preverbal behaviors, vocabulary development, social referencing and verbal comprehension, preverbal communication, vocabulary development, and morphosyntactic development (Borrero et al. 2022). Their motor skills generally consist of motor planning, motor speed, perceptual–motor integration, functional hand skills, and motor skills, primarily in the limbs and torso, such as locomotion, coordination, balance, and motor planning (Bayley 2006).

There is currently a limited understanding of neurodevelopmental, social–emotional, and environmental (including socioeconomic and racial, ethnic, and health disparities) factors concerning children’s high risk of developmental delays and their consequences in the future (McLester-Davis et al. 2021). Therefore, the early identification of children at risk of neurodevelopmental disabilities may increase access to intervention, potentially improving the outcome (Kvestad et al. 2022). The WHO recognizes neurofunctional evaluation using the International Classification of Disability and Health as a valid developmental screening tool, which is gaining popularity for the assessment of development (World Health Organization 2007). For neurodevelopmental evaluation, the use of the Bayley Scales of Infant and Toddler Development—third version (BSID-III, now Bayley 4) is also widely accepted around the world (Hoskens et al. 2018). Researchers have used the BSID-III to evaluate neurodevelopment in preterm children for the purpose of EI qualification and intervention (Atkins et al. 2019; Borrero et al. 2022; Klein-Radukic and Zmyj 2023; Long et al. 2012; Perra et al. 2015).

### 1.3. Assessment of Children’s Mastery Motivation and Neurodevelopment

Concerning the assessment of preterm children’s mastery motivation, studies (Barrett et al. 2017; Busch-Rossnagel and Morgan 2013; Józsa and Molnár 2013; Morgan et al. 2017b) have suggested two means of assessment, i.e., the use of the DMQ18 and individualized challenging behavioral tasks. Several ways of assessing children’s mastery motivation have been implemented in studies (e.g., by observing the context of children’s free play; Jennings et al. 1988), such as the use of structured challenging tasks (Barrett et al. 2017; Józsa et al. 2017), the application of rating scales by parents or teachers (Hwang et al. 2017; Józsa and Molnár 2013; Morgan et al. 2017b), and the use of self-rating scales by children (Józsa and Morgan 2014, 2017; Józsa et al. 2014). 

According to Morgan et al. (2020), the DMQ18 considers six factors of children’s mastery-related behaviors based on adults’ perceptions. Out of these six factors of the DMQ18, the first four factors are included to measure the instrumental context of children’s cognitive/object-oriented persistence, gross motor persistence, and social persistence with their peers or adults. The remaining two factors assess expressive aspects of mastery motivation, such as mastery pleasure and negative reactions to the failure of mastery motivation. Morgan et al. (2020) claimed that the DMQ18 can also measure children’s skills with respect to mastery tasks, such as general competence. Different versions of the DMQ18 are available because children of different ages face different levels of difficulty and challenges (Green and Morgan 2017). 

The findings of one Italian study on neurodevelopmental skills (Gasparini et al. 2017) showed that Italian preterm infants’ performance level was significantly lower than that of their full-term peers, with the exception of their expressive language and gross motor subscales. In the investigation of the predictive value of the BSID-III with respect to the development of very preterm/very low-birth-weight children, one study (dos Santos et al. 2013) found that children’s motor scale scores on the Bayley scale had a moderate predictive value with respect to their later motor functions. Furthermore, the BSID-III is still widely used in follow-up clinics that serve as a primary referral source to EI (Blasco et al. 2020; Tang et al. 2012; Wang et al. 2017). It is also the most commonly applied method for assessment of children’s developmental aspects. The BSID-III measures five main factors of children’s developmental aspects, i.e., as cognitive, expressive language, receptive language, fine motor, and gross motor skills. 

### 1.4. Predictions for Early Intervention (EI) 

Multiple researchers have reported chronic underenrollment of children born LBW/preterm in EI programs in the United States (Atkins et al. 2017, 2019; Blasco et al. 2017; Hebbeler et al. 2007; Tang et al. 2012). Previous research indicates that preterm children who exhibit lower scores on developmental assessments and more severe medical indications, such as younger gestational ages and longer hospital stays in the NICU, are more likely to be enrolled in EI programs (Atkins et al. 2017; Litt and Perrin 2014; Tang et al. 2012). Age at testing and birth weight have also been found to be significant predictors of EI attendance (Blasco et al. 2017). Psychosocial factors (e.g., education level and income) also play a mediating role in the utilization of services such as EI (Hoffman et al. 2015).

The increasing number of preterm infants has created a trend of children who are at high risk for developmental delay and who would subsequently benefit from EI services, including speech therapy, physical therapy, and/or other types of services based on the child’s and family’s needs (Martin et al. 2018). Unfortunately, traditional standardized assessment measures often fall short of identifying cognitive and social delays in children born with LBW/preterm that could assist in EI qualification and enrollment (Blasco et al. 2017; Keilty et al. 2015). Spencer-Smith et al. (2015) found that cognitive and language scores on the BSID-III did not predict later impairments in these areas. Additionally, there has been significant concern that the corrected age on the BSID-III overestimates a child’s cognitive and developmental abilities (Acton et al. 2011), and it has been found to be a poor predictor of later academic performance (Anderson and Burnett 2017). Hence, it is crucial to evaluate how effective mastery motivation is in terms of contributing to the overall evaluation of young children who have experienced developmental delays. 

### 1.5. Research Aims and Questions

The literature cited above demonstrates that some researchers used the DMQ18 only (e.g., Green and Morgan 2017; Hwang et al. 2017; Morgan et al. 2017b; Wang et al. 2011), and some researchers applied only BSID-III tests (e.g., Bayley 2006; Kvestad et al. 2022; Tang et al. 2012; Wang et al. 2017) for the assessment of children’s neurodevelopment. Therefore, it is necessary to assess the relationship between preterm children’s DMQ18 scale scores and their BSID-III test scores and to investigate differences between infants and toddlers among these two types of measurements.

Second, for young children at high risk of developmental delays, EI services are crucial for their developmental process. Therefore, it is also essential to investigate predictors (e.g., mastery motivation, other developmental processes, parents’ education level, and birth weight), which are important for identifying the early intervention engagement for these high-risk children.

Based on these aims, we plan to address the following research questions:

1. What is the relationship between preterm children’s (infants/toddlers’) DMQ18 scale scores and BSID-III scale scores based on LBW and VLBW?
2. What are the differences between preterm infants and toddlers born with LBW (<2500 g) or VLBW (<1500 g) in terms of their DMQ18 and BSID-III scores?
3. What are the predictors for the early intervention of young high-risk children with developmental delays?

## 2. Methods

### 2.1. Design and Procedure

In this study, we investigated preterm infants and toddlers aged between three months and three years old who were born with LBW and VLBW. These participants were seen in a high-risk infant follow-up clinic located in a major metropolitan area in the Pacific Northwest region of the United States. Children were included in the study if they were seen in the clinic between May 2015 and December 2018. Children with a known genetic disorder and/or previously diagnosed disabilities such as cerebral palsy attended another clinic. Demographic and medical information was obtained from a parent-completed clinical intake form, including gender, number of siblings, and home environment (single, two parents, relatives (e.g., grandmother)), as well as the qualification and attendance in early intervention.

Children completed a standardized infant/toddler developmental assessment (i.e., BSID-III), while parents completed a Likert questionnaire on mastery motivation (DMQ18). The DMQ18 was provided to parents in either English or Spanish, depending on the family’s preferred language. Hospital-qualified interpreters were used as part of routine clinical care when a child and/or family did not speak English as a first language. In this study, we utilized data from a larger clinical dataset approved by the organization’s Institutional Review Board. The study was completed in accordance with prevailing ethical standards. All private medical information (child and parent names and medical identification numbers) was removed prior to data analyses. 

### 2.2. Participants

Children in the study (N = 233) ranged from a chronological age of 6.87 months to 39.03 months (*M* = 14, SD = 8.23) at the time of their assessment. The total sample was divided into an infant group (*n* = 178; *M* = 10.0 months) and a toddler group (N = 55; *M* = 26.7 months). Of those children, 209 had scores corrected for prematurity, as specified in the BSID-III administration manual (Bayley 2006; range five months to 23.9 months). The remaining children were over 24 months of age, so the BSID-III scores were based on chronological age. The gestational age for the sample ranged from 23.57 to 36.86 weeks (*M* = 32, SD 2.92). The average birth weight was 1666 g (range: 415 to 2470 g; SD: 498 g). We also categorized preterm children into two groups based on their birth weights: those with very low birth weight (VLBW), weighing < 1500 g (N = 133, M = 1006 g, SD = 278 g), and those with low birth weight (LBW), weighing > 1500 g and < 2500 g (N = 100, M = 2059 g, SD = 372 g). The groups of participants are also displayed within Table 1 for enhanced visibility. Over half (57%) of the children were male. Type of insurance (public vs. private) was used as a proxy for socioeconomic status; over half (52%) of the sample had public insurance (n = 113). Of the 219 participants that provided a response to a question regarding EI, 75 (34%) were receiving EI support at the time of their appointment. For additional demographic information, please refer to Table 2.

### 2.3. Measures

#### 2.3.1. Revised Dimensions of Mastery Questionnaire (DMQ18)

The revised DMQ18 has been used worldwide to assess mastery motivation (Morgan et al. 2017b). The current DMQ18 was revised in the fall of 2014 and is available in English, Chinese, Hungarian, and Spanish (Morgan et al. 2020). The revised DMQ18 has three age-based versions (infant—6 to 18 months, preschool age—19 months to 5 years, and school-age—over 5 years) (Green and Morgan 2017). For this study, the infant version of the revised DMQ18 was used for infants, and the preschool version was used for toddlers. Of the sample, 89.5% completed the English revised DMQ18, and 10.5% completed a revised Spanish DMQ18. We used the revised DMQ18 to ask a parent and/or teacher to rate their perception of the child’s behavior from 1 (“not at all like this child”) to 5 (“exactly like this child”). 

The revised DMQ18 infant version comprises thirty-eight items that measure seven dimensions of mastery motivation: (1) cognitive/object persistence (6 items), (2) gross motor persistence (5 items), (3) social persistence with adults (6 items), (4) social persistence with children (6 items), (5) mastery pleasure (5 items), (6) negative reactions to challenge—frustration/anger (5 items), and (7) general competence (5 items). 

**Table 2:** Demographic information (N = 233).

Variable	N	Percent
Sex		
Male	132	56.7
Female	101	43.3
Race/ethnicity * (N = 181)		
Caucasian	116	64.1
African American	7	3.9
American Indian	1	0.6
Native Hawaiian or Pacific Islander	4	2.2
Asian American	12	6.6
Hispanic	27	14.9
Multiple ethnicities	14	7.7
Mother’s education * (N = 198)		
Some high school	23	11.6
High school graduate	43	21.7
Some college	49	24.7
College graduate	51	25.8
Post college	32	16.2
Father’s education * (N = 190)		
Some high school	37	19.5
High school graduate	36	18.9
Some college	51	26.8
College graduate	47	24.7
Post college	19	10.0
Insurance (N = 216)		
Public	113	52.3
Private	103	47.7
Language (N = 233)		
English	198	86.5
Spanish	24	10.5
Other	7	3.0

* Note: Percentages are based on valid responses. Information on demographic variables is missing for some respondents.

The revised DMQ18 toddler (preschool) version, and (7) general competence measures the seven dimensions of mastery motivation listed above. However, as toddlers can describe sadness or shame, we divided the dimension of negative reaction to challenge—frustration/anger into two parts: negative reaction to frustration—anger and negative reaction to frustration–sadness/shame, creating eight total domains in the 39-item toddler version: (1) cognitive/object persistence (5 items), (2) gross motor persistence (5 items), (3) social persistence with adults (5 items), (4) social persistence with children (6 items), (5) mastery pleasure (5 items), (6) negative reaction to frustration—anger (4 items), (7) negative reaction to frustration—sadness/shame (4 items), and (8) general competence (5 items). Table 3 provides an example of DMQ18 items for both the infant and toddler versions.

#### 2.3.2. Bayley Scales of Infant and Toddler Development-III

The BSID-III is a norm-referenced tool that measures cognitive, expressive language, receptive language, fine motor, and gross motor skills for children between the ages of 16 days and 42 months (Bayley 2006). The scale provides composite scores with a mean of 100 and a standard deviation of 15 for the cognitive, language, and motor scales. Scaled scores for the cognitive, receptive, and expressive language, as well as the fine and gross motor subtests, have a mean of 10 and a standard deviation of 3. 

#### 2.3.3. Assessment for the Qualification of Early Intervention Enrollment

We applied one dichotomous item (“Yes” or “No”) to assess whether preterm children are qualified to enroll in an EI program. 

## 3. Results

### 3.1. Reliability, Validity, and Homogeneity Measures of Instruments

For the internal consistency reliability of instruments, we analyzed the scale reliability using Cronbach’s alpha. The DMQ18 measures of both infant and toddler versions (r = 0.81 and r = 0.83) were acceptable to address our research questions (Morgan et al. 2020). The means and standard deviation of the infant version of the DMQ were 3.39 and 0.73, respectively, while for toddlers, they were 4.24 and 0.12, respectively. The infant version of the DMQ follows a normal distribution, as evidenced by the acceptable values of skewness (−0.59) and kurtosis (−0.38). Similarly, the toddler version also conforms to a normal distribution, with a skewness value of −0.84 and a kurtosis value of −0.22. For the BSID-III measures of assessing children’s mastery tasks, the internal consistency reliability values of the infant and toddler versions are 0.89 and 0.88, respectively (see Table 4). The mean, standard deviation, skewness, and kurtosis of the infant version of the BSID-III measure are 8.59, 2.51, 0.30, and 0.36, respectively, demonstrating that the test follows a normal distribution. Similarly, the toddler version of the BSID-III measure also exhibits acceptable values for the mean (8.11), standard deviation (2.47), skewness (0.19), and kurtosis (0.55), indicating that the test is in accordance with a normal distribution.

Investigating the instrument (DMQ18) developers’ studies (Józsa and Barrett 2018; Józsa and Morgan 2014; Józsa et al. 2014, 2019; Morgan et al. 2017a, 1990, 2020), it was found that the DMQ18 instrument was reliable and valid according to test–retest reliability and parallel-forms reliability measures. The test–retest reliability of the DMQ18 rated by parents varied between r = 0.70 and r = 0.97, and parallel-forms reliability ranged between 0.72 and 0.87. In terms of content validity, the DMQ18 had a strong foundation (starting in the 1980s), with different versions implemented in some studies (Jennings et al. 1988; Morgan et al. 1990). Infant and toddler versions have been translated and validated in five different languages: Hungarian, Turkish, Chinese, Spanish, and Persian (Shaoli et al. 2019). In order to determine the content validity when administered in different languages, some studies have already validated the DMQ18 English version with experts input (e.g., using the Bangla version by Shaoli et al. 2019), applying the Indonesian version by Rahmawati et al. (2020), and using the Swahili version on Kenyan preschool children by Amukune et al. (2021). The experts consulted in these studies also approved the content validity of the DMQ18. In this study, we also proved the criterion validity of the DMQ18 in terms of concurrent evidence and predictive evidence. For concurrent evidence, our DMQ18 versions are closely related to the standardized test (BSID-III) of children’s developmental scales, which can be used as a criterion for testing children’s behavioral mastery tasks (Huang and Lo 2019). Huang and Lo’s (2019) study showed that children’s DMQ18 social persistence, total persistence, and total mastery motivation were significantly and positively related (rs = 0.25–0.27, ps < .05) to the global motivation scales of young children. For predictive evidence, the DMQ18 was used to examine the effectiveness of mobility intervention in a randomized control for preterm children with developmental delays (Huang et al. 2022). 

With respect to the reliability of the BSID-III, we investigated the test developers’ studies (Bayley 1949, 2006, 2014) through split-half and test–retest stability measures. The split-half reliability coefficients for all subtests and scales ranged from 0.71 to 0.98, whereas the test–retest stability coefficients varied between 0.80 and 0.87. The validity of the BSID-III test was also investigated through a series of studies (Bayley 1949, 2006, 2014; Krogh and Væver 2019) focusing on the intercorrelations between the subtests, the factor structure of the test, correlation with other measures, and special group studies. These studies provided insights, showing that the subtests of the BSID-III were correlated with one another and that the hypothesized models fit the data best. Moreover, the test correlations were also consistent, as expected. It was also confirmed that the BSID-III test was valid as a way to differentiate healthy children from those at high risk (Bayley 2006). 

### 3.2. DMQ18 Homogeneity Measures for Both Infants and Toddlers

**Figure 1:**
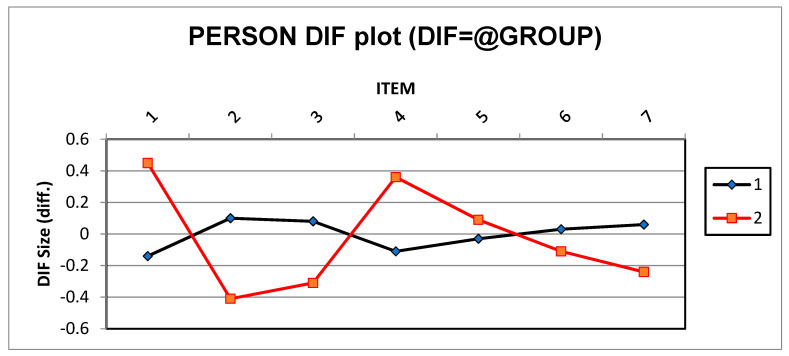
Differential item functioning for both infant and toddler versions of the DMQ18. Note: 1 (infant version), 2 (toddler version).

Differential item functioning (DIF) analysis was conducted to check whether there were item biases based in the versions (infant or toddler) of the DMQ18. DIF analysis suggested participant responses based on the version for each item of measuring children’s mastery motivation (Oo et al. 2023a). Categorization of DIF analysis results into three categories was recommended by Oo et al. (2023a), namely negligible, slight to moderate (DIF ≥ 0.43 logits), and moderate to large (DIF ≥ 0.64 logits). DIF analysis (see Figure 1) showed that none of the items from either version had biased measures (DIF = 0.45 logits). DIF logits in the toddler version were slightly higher than the recommended values (Oo et al. 2023b), possibly because the toddler version included two more factors, i.e., negative reactions—frustration/anger and negative reactions—sadness/shame. 

The normality of the DMQ18 measures was also confirmed between both infant and toddler versions. As a result, the overall skewness values of the infant and toddler versions were −1.99 and 2.32, and their overall kurtosis scores were −0.04 and −0.02, respectively (consistent with Kline’s (2011) recommendation for violation values of Sk > 0.3 for skewness and K > 0.10 for kurtosis). These values suggest that all variables were relatively normally distributed. Based on the above results, we confirmed that our instruments were homogeneous for both infant and toddler versions, making them suitable to address our research questions. 

### 3.3. BSID-III Homogeneity Measures for Both Infants and Toddlers

**Figure 2:**
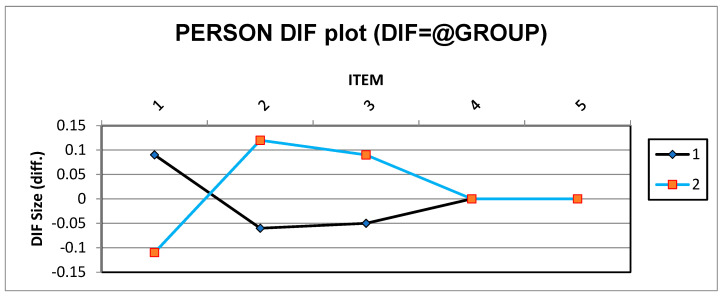
Differential item functioning for both infant and toddler versions of the BSID-III. Note: 1 (infant version), 2 (toddler version).

Figure 2 shows the acceptable logit numbers of the infant and toddler versions (DIF logits between −0.15 and 0.15) of the BSID-III measurement (Oo et al. 2023b). Therefore, we can assume that there are no biased measures in either the infants or toddler version of the BSID-III. The normality measures of skewness and kurtosis were 1.66 and 0.19 for the infant version, and 0.03, and 0.05 for the toddler version, respectively. Therefore, we can say that both BSID-III tests were homogenous and normal, making them suitable to measure preterm children’s mastery motivation (based on individualized behavioral tasks).

### 3.4. Addressing RQ_1_

All statistical data were analyzed using SPSS software. We used correlations to examine the relationships between the Bayley-III and the DMQ18. Multivariate tests were used to compare children born with VLBW and those born with LBW. For the infant group, there were relatively few significant correlations (14%; Table 5). DMQ18 gross motor persistence and general competence were significantly related to the BSID-III gross motor scale. DMQ18 general competence and negative reaction to challenge—frustration/anger were significantly related to the BSID-III fine motor scale. Finally, DMQ18 social persistence with children was significantly related to the BSID-III cognitive scale.

Among the toddler groups, there was an increased number of significant correlations (33%; Table 6). Specifically, DMQ18 cognitive/object persistence, gross motor persistence, negative reaction—sadness/shame, and general competence were significantly related to the BSID-III receptive language scale. DMQ18 cognitive persistence/object persistence, mastery pleasure, and general competence were significantly related to the BSID-III expressive language scale. Over half of the DMQ18 scales (i.e., cognitive/object persistence, gross motor persistence, mastery pleasure, negative reaction—sadness/shame, and general competence) were significantly related to the BSID-III cognitive scale. 

### 3.5. Addressing RQ_2_

To address this research question, multivariate analysis was used to assess whether there were overall differences between LBW and VLBW infants on the DMQ18 scales; this test was statistically significant (*F* (7, 167) = 2.38, *p* = .024), with a medium to large effect size (η^2^ = 0.091) according to Cohen (1988). Follow-up univariate ANOVAs indicated that infants born with VLBW scored significantly higher (showed more frustration/anger) on negative reaction to challenge—frustration/anger, with a medium effect size. Infants born with VLBW scored significantly higher on the DMQ18 negative reaction than item than infants born with LBW (Table 7).

A subsequent multivariate analysis was used to assess differences on BSID-III scales between infants born with LBW and VLBW, which were found to be statistically significant (*F* (5, 171) = 5.28, *p* < .001), with a large to medium effect size (η^2^ = 0.134). Follow-up univariate ANOVAs indicated that infants born with VLBW scored statistically significantly lower on the BSID-III cognitive, receptive language, fine motor, and gross motor scales, with small to medium-sized effects (Table 8). There was not a significant difference found between infants born with LBW and VLBW on the BSID-III expressive language scale. 

Infants born with VLBW scored significantly lower on the BSID-III cognitive, receptive language, fine motor, and gross motor items than infants born with LBW.

We conducted the same multivariate analysis to assess whether there were differences between toddlers born with LBW and VLBW on the BSID-III scores; this test was statistically significant (*F* (5, 40) = 2.71, *p* = .034, η^2^ = 0.253), with a large effect size. Follow-up univariate ANOVAs indicated that toddlers born with VLBW scored statistically and significantly lower on the cognitive, expressive language, and fine motor BSID-III scales (Table 9), with medium to large effect sizes for the significant ANOVAs. There were no significant differences found between toddlers born with LBW and VLBW on the BSID-III receptive language and gross motor scales. A subsequent multivariate analysis was used to assess differences on DMQ18 scales for toddlers born with LBW and VLBW; this test was not statistically significant (*F* (8, 39) = 1.77, *p* = .11). Therefore, univariate F values are not reported.

Toddlers born with VLBW scored significantly lower on the BSID-III cognitive, expressive language, and fine motor scales than toddlers born with LBW. 

### 3.6. Addressing RQ_3_

Binary logistic regression was conducted to examine whether five predictor variables, i.e., birth weight, number of siblings, mother’s education, father’s education, and home living environment (mother only, father only, mother and father, other relatives, two mothers, and other), significantly predicted whether or not preterm children would enroll in EI. When all five variables were considered together, they significantly predicted whether or not the preterm children would enroll in EI (χ^2^ = 8.125, df = 5, N = 233, *p* < 0.05). Table 10 presents the odds ratios (Exp(β)), which suggest that the odds of correctly estimating who will enroll in EI improve by 40% if one knows preterm children’s birth weight and by 36% if one knows children’s home environmental status (i.e., mother only, father only, mother and father, other relatives, two mothers, or other).

**Figure 3:**
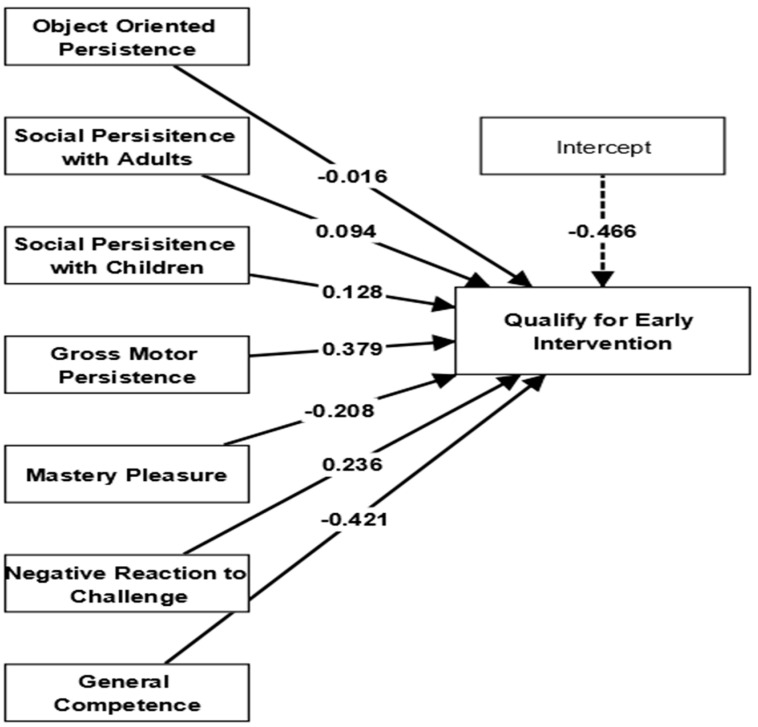
Impacts of infants’ mastery motivation on early intervention qualification.

In addition to examining the predictors mentioned earlier, we also assessed the effectiveness of evaluating mastery motivation to determine whether preterm children are eligible for EI programs. As a result, we fitted the logistic regression model using SmartPLS software to develop models of association that can forecast the factors that impact preterm children’s mastery motivation in relation to their suitability for enrollment in EI programs. In this logistic regression analysis, the outcome variable was the qualification for EI (represented on a binary scale), while the predictor variables included ‘object-oriented persistence, social persistence with adults, social persistence with children, gross motor persistence, mastery pleasure, negative reaction to challenge, and general competence’. The logistic regression model was set up with a two-tailed test, a significance level of .05, a maximum of 1000 iterations, and a stop criterion of 10^−5^ (accurate). Subsequently, the calculations were initiated. Findings showed that the mastery motivation of infants with respect to their eligibility for EI had a good fit for the accurate interpretation, as evidenced by lower values of the Akaike information criterion (AIC = 248.40) and Bayesian information criterion (BIC = 251.58), as well as Nagelkerke’s R-square value of 0.46. Consequently, the study results suggest that a child’s social persistence with other children (β = 0.128, SE = 0.309, * *p* < .05), gross motor persistence (β = 0.379, SE = 0.201, * *p* < .05), and negative reaction to challenge (β = 0.236, SE = 0.332, * *p* < .05) are significant factors in determining their eligibility for early intervention programs (as shown in Figure 3).

**Figure 4:**
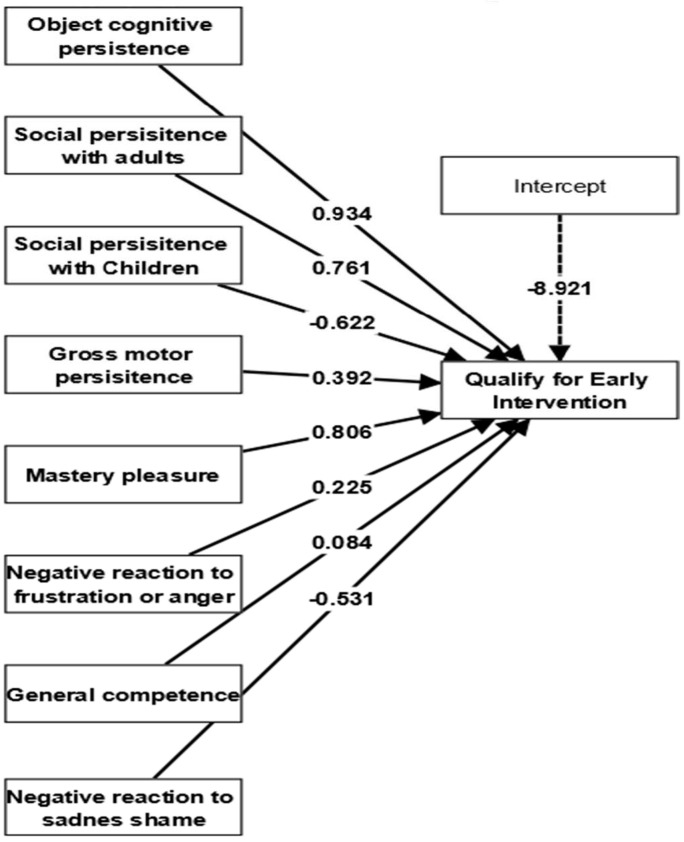
Impacts of toddlers’ mastery motivation on early intervention qualification.

The effectiveness of toddlers’ mastery motivation in determining their eligibility for EI enrollment was also examined. In this logistic regression model for toddlers’ mastery motivation, EI qualification (serving as the dependent variable) was influenced by eight predictor variables: object cognitive persistence, social persistence with adults, social persistence with children, gross motor persistence, mastery pleasure, negative reaction to frustration or anger, negative reaction to sadness or shame, and mastery motivation. Similar to the above description, the logistic regression model was established with a two-tailed test, a significance level of .05, a maximum of 1000 iterations, and a stop criterion of 10^−5^ (accurate). Subsequently, the calculations were initiated. The logistic regression model showed a good fit, as indicated by the lower values of AIC (248.40) and BIC (251.39), as well as Nagelkerke’s R-square value of 0.55. The study results suggest that toddlers’ objective cognitive persistence (β = 0.934), social persistence with adults (β = 0.761, SE = 0.441, * *p* < .05), gross motor persistence (β = 0.392, SE = 0.375, * *p* < .05), mastery pleasure (β = 0.806, SE = 0.334, * *p* < .05), and negative reaction to frustration/anger (β = 0.225, SE = 0.582, * *p* < .05) had significant impacts on predicting for their EI enrollment qualification (as depicted in Figure 4). 

## 4. Discussion

Our study measured infants’ and toddlers’ mastery motivation and neurodevelopment in order to identify young children at high risk of developmental delays. We applied two main types of measurement (e.g., the DMQ18 and BSID-III) to investigate the relationships between infants’ and toddlers’ mastery motivation and neurodevelopment, as well as differences between infants and toddlers born with LBW (<2500 g) and VLBW (<1500 g) on the DMQ18 and BSID-III scores. Different variables from children’s background information (e.g., birth weight, number of siblings, father’s education, mother’s education, and home environment) were also measured to determine which variables predicted their EI enrollment. Before addressing our research questions, first, we confirmed the reliability, validity, and homogeneity measures of our instruments. Internal consistency, reliability, skewness, kurtosis measures, and Rasch analyses proved that our instruments were reliable and homogeneous, making them suitable to address our research questions.

We found significant relationships between the DMQ18 and BSID-III; results revealed that the DMQ18 contributed meaningfully to the overall assessment of infants and toddlers. It is not surprising that DMQ18 general competence was significantly related to both BSID-III gross and fine motor skills at the infant level, as motor items are often most pertinent to infant developmental assessments. Likewise, at the toddler age, DMQ18 cognitive/object persistence and general competence were significantly related to BSID-III cognitive, expressive, and receptive language subscales. This may be the result of a toddler’s increased opportunity to demonstrate skills to their parents, as well as greater item density on the BSID-III, particularly for cognitive and motor skills at the toddler age. Wang et al. (2016) similarly found significant correlations between cognitive/object persistence ratings on the DMQ18 for toddlers who were developmentally delayed. These correlations demonstrate the relevance of parents’ reports in contributing to an overall view of a child’s ability and to provide support, including the DMQ18 in an EI “toolbox”, as it can reliably be used with children who have developmental delay and children born with LBW/preterm.

In this study, we also found a significant negative correlation between infant scores on the DMQ18 negative reaction to challenge–frustration/anger and the BSID-III fine motor scale. This is not surprising, as an infant who is competent with fine motor tasks is less likely to become frustrated when he/she is not immediately successful. Indeed, these fine motor abilities are some of the earliest persistent abilities demonstrated in developmental assessments (Barger et al. 2018). Negative reaction to challenge—frustration/anger is important, as it has been shown to be predictive of later emotional and cognitive development (Józsa and Barrett 2018). Niccols et al. (2003) proposed a bidirectional relationship between motivation and competence; if the child is more successful in attempts, he/she is more motivated to try, which makes sense, given the present findings. Therefore, interventionists should assist parents, care providers, and specialists who interact with infants and toddlers at increased risk of developmental disabilities to facilitate an early mastery of motivational skills. These findings have worldwide implications for EI, given previous international studies and successful translations of the DMQ18 (Liao et al. 2021).

Multivariate analyses of score differences revealed that infants born with VLBW scored lower than their peers with heavier birth weights on both the DMQ18 negative reaction to challenge—frustration/anger and all scales of the BSID-III except the expressive language subscale. This finding is not surprising, as there is low item density for expressive language on the BSID-III, especially during the infant years (Blasco et al. 2020). The mean age of infants included in this study was 10 months. In contrast, the expressive language subscale was significant for toddlers, which indicates that, similar to previous research, children born with VLBW are more likely to have trouble with emotional regulation, which can lead to lifelong struggles and at-risk behavior. In one study (Zablotsky et al. 2019), it was also found that there were significant differences in developmental delay for boys, children with birth weight ≥2500 g, children living in urban areas, and those with less-educated mothers. In the toddler group, although significant relationships were found between the DMQ18 and the BSID-III scales, there were no differences based on birth weight on the parents’ DMQ18 report. This finding may indicate that parents rated their children similarly despite their birth weight. This was the only incongruence between the parent report and the BSID-III, as children born with VLBW were rated lower on the BSID-III. 

In terms of predictive modeling for EI enrollment, our regression model indicated that the enrollment for both infants and toddlers was predicted by the combined factors of birth weight, number of siblings, father’s education, mother’s education, and children’s home environment. Among these combined factors, preterm children’s birth weight and their home environment were found to be significant predictors of early intervention enrollment. Since our study has highlighted the significance of the family environment, we can support families through early intervention partnerships with infant/toddler mental health and social work partners. These results are in line with those reported in other studies (Atkins et al. 2017; Blasco et al. 2017; Litt and Perrin 2014; Tang et al. 2012) indicating that the lower the birth weight, the more likely the infant will be enrolled in EI services. Barger et al. (2018) also found birth weight to be a very common prevalent category predicting EI eligibility, used in 19 states in the United States. Wang et al.’s (2011) study also showed that the home living environment had moderate relationships with toddlers’ mastery motivation. Furthermore, parental support and toy responsiveness are crucially important for toddlers’ mastery motivation and EI engagement (Banerjee and Tamis-LeMonda 2007). Parental scaffolding of early mastery attempts has also been shown to promote a child’s persistence and later competence at school age (Prendergast and MacPhee 2018).

Finally, our study investigated whether infants’ and toddlers’ mastery motivation could contribute to the battery of assessments for EI enrollment. It was found that both infants’ and toddlers’ mastery motivation had a significant predictive effect on their EI engagement. Specifically, infants’ social persistence with other children, gross motor persistence, and mastery pleasure, as well as toddlers’ objective cognitive persistence, social persistence with adults, gross motor persistence, mastery pleasure, and negative reaction to frustration/anger, are significant factors that seem to influence eligibility for early intervention programs. This means that preterm children’s mastery motivation assessment by the DMQ18 is of considerable importance in predicting their EI enrollment. Our study results are line with those of a previous study (Huang et al. 2022) that suggested the importance of parents’ role and that mastery motivation of toddlers is necessary for EI programs. In our study, we found that the traditional standardized BSID-III assessment measures were not able to predict the enrollment of preterm children in EI programs. As a result, we excluded these insignificant findings and instead focused on the significant results obtained from the prediction values using mastery motivation measured by the DMQ18, along with five other predictor variables: birth weight, number of siblings, mother’s education, father’s education, and home living environment. Our findings also suggest that the traditional standardized assessment BSID-III measures cannot predict preterm children’s EI enrollment (Blasco et al. 2017; Keilty et al. 2015) in isolation. The findings of our study revealed that both children’s birthweight and the quality of their home environment emerged as significant predictors for their enrollment in EI programs. The factors influencing the prediction effects on EI enrollment may also be influenced by additional predictor variables. In contrast to the findings of previous researchers (Atkins et al. 2017, 2019; Blasco et al. 2017; Hebbeler et al. 2007; Tang et al. 2012) that demonstrated underenrollment of children born with low birthweight (LBW) in EI programs in the United States, our study yielded different results in terms of predicting the impact of birthweight on EI enrollment.

## 5. Limitations

In the present study, we utilized parent report data for the DMQ18 measure, which may include response bias. However, researchers using the DMQ18 reinforce the importance of parental reports and indicate that parents may be more accurate reporters of their child’s cognitive and social persistence than traditional measures, such as the BSID-III (Wang et al. 2011). Indeed, parent reports have been found to match well with behavioral developmental outcome measures at the toddler age (Wang et al. 2011). In our study, we used two language versions of the DMQ18 (English and Spanish), which may raise some cultural differences. However, both versions were previously translated into English and Spanish and were content-validated (Shaoli et al. 2019). Despite these limitations, this study included an assessment of the historically studied population, which provides useful information on our current DMQ18 and BSID-III measures in predicting EI enrollment. Our main emphasis was on children receiving medical care at the clinic, and we did not include data from the broader population of healthy children. Consequently, we acknowledge that excluding healthy children may have introduced sample bias, which could have potentially inflated the correlation between the two variables of the DMQ18 and BSID-III measures. 

Another limitation may be that at the time of BSID-III testing, a majority (90%) of the sample was corrected for prematurity in accordance with administration guidelines, which may have contributed to a lack of significant differences. This may be particularly true for the BSID-III at the infant age (Blasco et al. 2017). There have been long-standing concerns that the BSID-III provides higher-than-anticipated scores when correcting for prematurity (Anderson et al. 2010; Duncan et al. 2015; Hack et al. 2005). In a later study, Blasco et al. (2020) found no differences in BSID-III scores for 6-month-olds born with LBW and between preterm and full-term peers. However, differences were noted in DMQ18 cognitive and social persistence. 

Finally, although we found significant differences in EI enrollment based on the home environment, it should be noted that this factor was based on who was living in the home at the time of the assessments, and it does not measure the home environment in terms of the quality of child materials and/or parent–child interaction in the home setting. This study could have been enhanced by including a measure of parent–child interaction or observation in the home environment with our EI partners. This would help us capture health disparities related to racial, ethnic, and cultural differences. 

## 6. Conclusions

Our study shows that measurement of mastery motivation and neurodevelopment was crucial for identifying young children at high risk of developmental delays. It also highlights the importance of mastery motivation as a predictor for EI programs. Thus, mastery motivation is an important developmental factor in young children and should be evaluated and promoted in early education, given its predictive nature for preacademic and academic skills (Józsa and Barrett 2018). The DMQ18 could be considered as part of early childhood and early intervention authentic assessment as a key component of a comprehensive evaluation that includes parent and provider observations and informed clinical opinion. Team members should consider goal-directed behavior and social persistence, core skills of mastery motivation, as critical skills to address during early intervention (Keilty et al. 2015). Mastery motivation remains an overlooked construct that is critical for the development of young children. This is particularly true for children who are at high risk of developmental delay and who ideally receive developmental assessments early in life to assist in appropriate planning and interventions. 

In conclusion, the DMQ18, a parent report, contributes meaningfully to the overall assessment of young children. Some significant correlations were also found among DMQ18 and BSID-III scaled scores (e.g., DMQ18 gross motor persistence and general competence were significantly related to the BSID-III gross motor scale) in the infant version. In the toddler version, DMQ18 cognitive persistence/object persistence, mastery pleasure, and general competence were significantly related to the BSID-III expressive language scale. Investigating the differences in mastery motivation among infants and toddlers, it was found that infants born with VLBW scored significantly higher than toddlers (showed more frustration/anger) on negative reaction to challenge—frustration/anger, with a medium effect size. No significant differences were observed between toddlers born with LBW and VLBW on the BSID-III receptive language and gross motor scales. 

## Figures and Tables

**Table 1:** Number of participants in groups.

Group	Very Low Birth Weight	Low Birth Weight	Total
Infants	95	83	178
Toddlers	38	17	55
Total	133	100	233

**Table 3:** Sample DMQ18 questions for each dimension rated by a parent.

Dimension	Infant	Toddler
Cognitive/object persistence	“Repeats a new skill until he or she can do it.”	“Works for a long time trying to do something challenging.”
Gross motor persistence	“Tries to do well in physical activities even when they are difficult.”	“Tries to do well in physical actives even when they are challenging.”
Social persistence with adults	“Tries to influence play with me or other adults.”	“Tries hard to get adults to understand him or her.”
Social persistence with children	“Tries to make other children feel better if they cry or seem sad.”	“Tries to say and do things that keep other kids interested.”
Mastery pleasure	“Claps hands or shows excitement when he or she is successful.”	“Gets excited when he or she figures something out.”
Negative reaction to challenge—frustration/anger	“Gets frustrated when not successful immediately.”	N/A
Negative reaction—frustration/anger	N/A	“Gets frustrated when not able to complete a challenging task.”
Negative reaction—sadness/shame	N/A	“Looks away when tries but cannot do something.”
General competence	“Does things that are difficult for children his or her age.”	“Does things that are difficult for children his or her age.”

**Table 4:** Reliability measures (Cronbach’s alpha) of instruments.

Instrument	Number of Domains		Version (Cronbach’s Alpha)		Total (Cronbach’s Alpha)
	Infants	Toddlers	Infants	Toddlers	
DMQ18	7	8	0.81	0.83	0.83
BSID-III	5	5	0.89	0.88	0.89

**Table 5:** Correlations between infants’ DMQ18 scale scores and BSID-III scaled scores.

Measure	Cognitive	Receptive Language	Expressive Language	Fine Motor	Gross Motor
Cognitive/object persistence	0.036	−0.091	0.030	−0.009	0.045
Social persistence with adults	0.076	−0.065	0.002	0.005	−0.007
Social persistence with children	0.153 *	0.021	−0.030	0.017	0.003
Gross motor persistence	0.049	0.045	0.115	0.085	0.188 *
Mastery pleasure	0.115	−0.060	0.043	0.008	0.075
Negative reactions to challenge—frustration/anger	−0.002	−0.105	−0.088	−0.151 *	−0.113
General competence	0.126	0.052	0.104	0.232 **	0.201 **

** Correlation is significant at the 0.01 level (two-tailed); * correlation is significant at the 0.05 level (two-tailed).

**Table 6:** Correlations between toddlers’ DMQ18 scaled scores and BSID-III scaled scores.

Measures	Cognitive	Receptive Language	Expressive Language	Fine Motor	Gross Motor
Cognitive/object persistence	0.419 **	0.529 **	0.330 *	0.222	0.339 *
Social persistence with adults	0.177	0.250	0.261	0.074	0.177
Social persistence with children	0.204	0.211	0.080	0.253	0.121
Gross motor persistence	0.342 *	0.412 **	0.201	0.242	0.228
Mastery pleasure	0.365 **	0.236	0.323 *	0.139	0.098
Negative reaction—frustration/anger	0.204	0.113	0.035	−0.051	0.179
Negative reaction—sadness/shame	0.377 *	0.363 *	0.252	0.265	0.167
General competence	0.420 **	0.469 **	0.330 *	0.241	0.277

** Correlation is significant at the 0.01 level (two-tailed); * correlation is significant at the 0.05 level (two-tailed).

**Table 7:** Differences between infants born with LBW and VLBW on the DMQ18 scales.

Measure	*df*	*F*	*p*	η^2^
Multivariate test	7.167	2.381	0.024	0.091
Cognitive/object persistence	1.173	0.309	0.579	0.002
Social persistence with adults	1.173	0.451	0.503	0.003
Social persistence with children	1.173	1.223	0.270	0.007
Gross motor persistence	1.173	0.764	0.383	0.004
Mastery pleasure	1.173	0.193	0.661	0.001
Negative reaction to challenge—frustration/anger	1.173	13.323	≤0.001	0.072
General competence	1.173	0.270	0.604	0.002

Note: *n* = 175.

**Table 8:** Differences between infants born with LBW and VLBW on the BSID-III scales.

Measure	*df*	*F*	*p*	η^2^
Multivariate test	5.171	5.275	≤0.001	0.134
Cognitive	1.175	6.269	0.013	0.035
Receptive language	1.175	13.652	≤0.001	0.072
Expressive language	1.175	2.490	0.116	0.014
Fine motor	1.175	19.288	≤0.001	0.099
Gross motor	1.175	8.642	0.004	0.047

Note: *n* = 177.

**Table 9:** Differences between toddlers born with LBW and VLBW on the BSID-III.

	*df*	*F*	*p*	η^2^
Multivariate test	5.40	2.708	0.034	0.253
BSID-III scales				
Cognitive	1.44	7.930	0.007	0.153
Receptive language	1.44	3.163	0.082	0.067
Expressive language	1.44	7.125	0.011	0.139
Fine motor	1.44	11.809	0.001	0.212
Gross motor	1.44	2.036	0.161	0.044

Note: *n* = 46.

**Table 10:** Logistic regression predicting who will enroll in early intervention (N = 233).

Variable.	β	SE	Wald	*p*	Exp(β), Odds Ratio
Birth weight	0.20	0.09	5.06	0.018 *	1.40
Number of siblings	0.94	0.12	0.61	0.434	1.10
Mother’s education	−0.08	0.08	1.06	0.304	0.92
Father’s education	0.01	0.08	0.02	0.895	1.01
Home environment	0.30	0.14	4.49	0.034 *	1.36

Note: * *p* < .05

## Data Availability

This research is based on human participants, and thus data availability is impossible due to their privacy.
